# Dataset of top-down nitrogen oxides fire emission estimation in northeastern Asia

**DOI:** 10.1016/j.dib.2022.108734

**Published:** 2022-11-09

**Authors:** Yuyun Fu, Rui Li, Jiheng Hu, Yipu Wang, Jiawei Duan

**Affiliations:** a State Key Laboratory of Fire Science, MEM Key Laboratory of Forest Fire Monitoring and Warning, School of Earth and Space Sciences, Comparative Planetary Excellence Innovation Center, University of Science and Technology of China, Hefei 230026, China; b Institut de recherche sur les forêts, Université du Québec en Abitibi-Témiscamingue (UQAT), Rouyn-Noranda J9 × 5E4, Canada

**Keywords:** Fire emission, Top-down approach, FRP, Nitrogen oxides, VIIRS

## Abstract

Fire emission is a major source of atmospheric nitrogen oxides (NO_x_ = NO_2_ + NO), accounting for a large part of global NO_x_ emission, which profoundly changes atmosphere physicochemical property and impacts human society. An effective evaluation of these impacts relies on accurate NO_x_ fire emission estimation. In this article, we developed a full top-down NO_x_ fire emission dataset for northeastern Asia based on the satellite-derived emission coefficient (EC) and fire radiative power (FRP) density. In the dataset, daily NO_x_ fire emissions during 2012–2019 were estimated at 1°x1° resolution across northeastern Asia, which can be used as fundamental input data in driving climate and weather models, and can be applied to investigate the characteristic of fire emission, fire-climate interaction, air pollution and human health effect. As a full top-down emission dataset, it can also serve as a reference for other existing emission inventories that are mostly based on bottom-up approaches.

**Specifications Table**

**Table:** 

Subject	Environmental Science
Specific subject area	Fire emission of trace gases
Type of data	Text
How the data were acquired	The top-down NO_x_ fire emission in this article was calculated by multiplying NO_x_ emission coefficient (EC, mass emission of per unit fire radiative energy released) with fire radiative energy (FRE). In the calculation, NO_x_ EC = 1.19 g/MJ for low-biomass vegetation fires, 0.41 g/MJ for forest fires, and 0.46 g/MJ for other vegetation fires were collected from the research article by Fu et al. (2022) entitled “Investigating the impacts of satellite fire observation accuracy on the top-down nitrogen oxides emission estimation in northeastern Asia”, in which NO_x_ EC was derived using near-simultaneous satellite observations of smoke NO_2_ concentration and active fire radiative power (FRP). As for FRE, it was computed based on FRP density from the public datasets—Global Fire Assimilation System (GFAS). More details can be found in the method Section 2.
Data format	Calculated
Description of data collection	Data of NO_x_ fire emission was computed from FRE and NO_x_ EC, where FRE was calculated using FRP density from GFAS, while NO_x_ EC was derived by Fu et al. (2022) entitled “Investigating the impacts of satellite fire observation accuracy on the top-down nitrogen oxides emission estimation in northeastern Asia”.
Data source location	The dataset covers the northeastern Asia. Spatial extent: longitude between 110°E and145°E; latitude between 40°N and 55°N.
Data accessibility	Repository name: Mendeley Data Data identification number: 10.17632/xbn3hj4mz7.4 Direct URL to data: https://data.mendeley.com/datasets/xbn3hj4mz7
Related research article	Fu, Y., Li, R., Hu, J., Wang, Y., & Duan, J. (2022). Investigating the impacts of satellite fire observation accuracy on the top-down nitrogen oxides emission estimation in northeastern Asia. Environment International, 169, 107,498. 10.1016/j.envint.2022.107498.

## Value of the Data

• The dataset provides fundamental input for climatic and atmospheric models.
• The dataset can be used to analyze the spatiotemporal variability of fire emission and impacts on air quality and human health.
• Most existing fire emission inventories are based on bottom-up approaches which rely on ground measurement. This dataset provides the full top-down NO_x_ fire emission estimation based on satellite observations, which can serve as a reference for bottom-up emission inventories and can be used to explore fire emission discrepancy among approaches.

## 1 Data Description

**Fig. 1:**
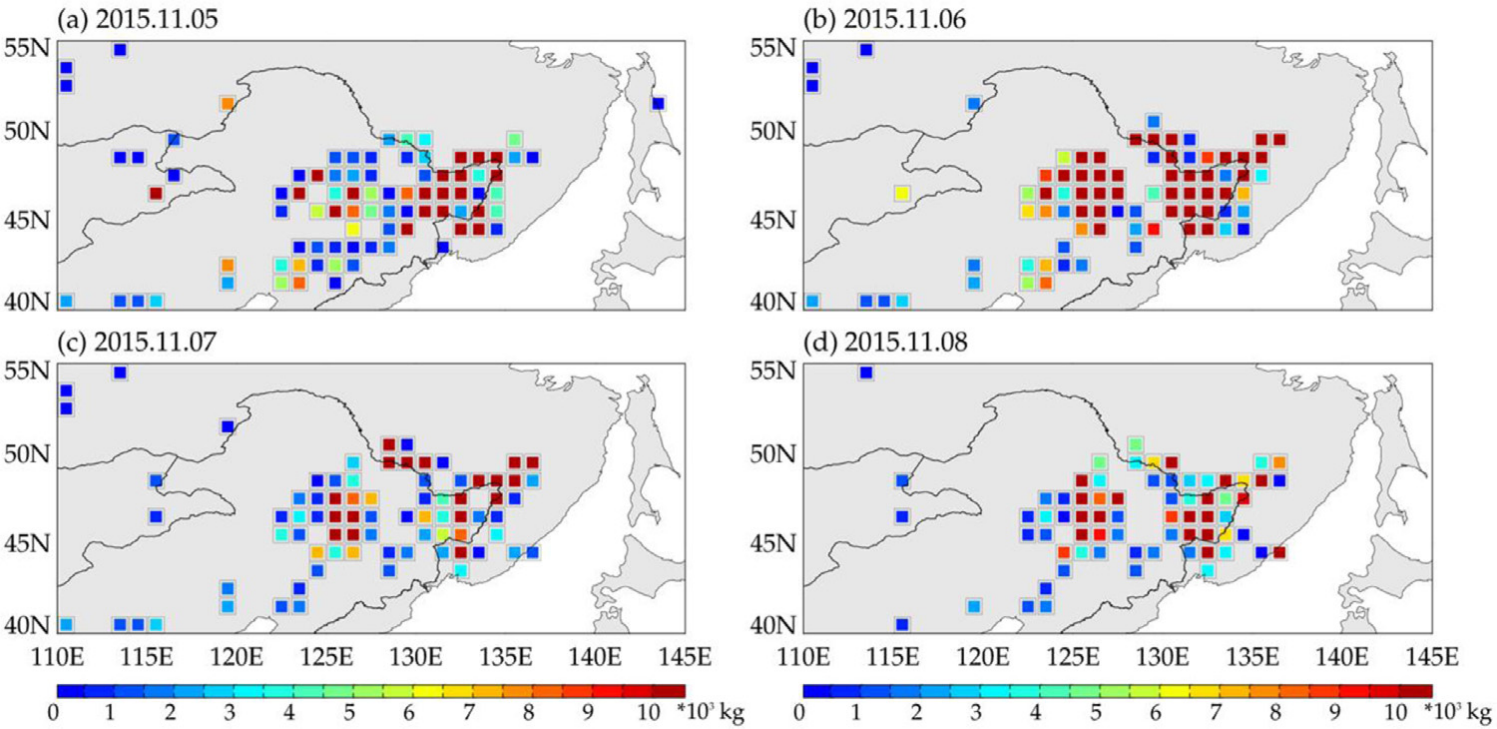
NO_x_ fire emission estimated for November 5th to November 8th in 2015 over northeastern Asia.

The dataset was produced using the method described in Section 2. It provides the full top-down estimation of NO_x_ fire emission at a temporal resolution of one day and spatial resolution of 1° in northeastern Asia (110 E–145 E; 40 N–55 N) during 2012–2019. Each data file is stored in the format of txt, and contains ID of grids, longitude and latitude in decimal degrees of grid center, and the total NO_x_ fire emission (unit, kg). Only those grids that have fire emission estimation are recorded. The name of data file is in the form as that: “NA.Full_Topdown_NOx_fire_emission.yyyymmdd.txt”, where “NA” indicates northeastern Asia, “yyyy” is fire year, “mm” is the month of year, and “dd” is the day of month. The size of each data file ranges from 1.0 kgbyte to 10 kgbytes (5.2 megabytes in total during 2012–2019), required very small memory to store, manage, and analyze. As an example, Fig. 1 presents the NO_x_ fire emission from the dataset during November 5th to November 8th in 2015.

The other materials that shared in the dataset are as follows:

(1) “VIIRS_EC_GFAS_FRE_daily_Emission_1 × 1_grid.pro”: It's the code file for the calculation of daily NO_x_ fire emission during 2012–2019 at 1° resolution over northeastern Asia, which is created using the programming language IDL (Interactive Data Language). Input data for the code includes the GFAS daily FRE and the daily FRP from VIIRS described as below.

(2) “VIIRS_daily_FRP_1 × 1_grids.rar”: There are data files that provide daily FRP from 2012 to 2019 in northeastern Asia with a spatial resolution of 1°, which is based on the 375-m active fire product VNP14IMG from VIIRS on Suomi-NPP satellite (see Section 2). Each file is stored in “.bin” format, and a structure with six structure variables are saved in file, namely “all_FRP”, “forest_FRP”, “low_FRP”, “other_FRP”, “lon”, and “lat”. Among them, the variables “all_FRP”, “forest_FRP”, “low_FRP”, and “other_FRP” are the total FRP (unit, MW) for all fires, forest fires, low-biomass vegetation fires, and other vegetation fires in the 1° x 1° grids, respectively; “lon” and “lat” are the longitude and latitude in decimal degrees of grid center. All structure variables are two-dimensional arrays, and each of them contains 35 (column) x 15 (row) elements. Missing value of “0.0″ is set for FRP if no fires are detected in a grid. Users can easily extract data in file use the methods that they are familiar with. For instance, one can restore and extract the FRP saved in a file via the following two IDL commands respectively:

Restore, “D:\VIIRS_daily_FRP_1 × 1_grids\2012\01\01.bin” forest_FRP_extracted = output.forest_FRP where “Restore” is IDL's restore procedure, “D:\VIIRS_daily_FRP_1 × 1_grids\2012\01\01.bin” is the data directory, the variable “forest_FRP_extracted” contains FRP of forest fires extracted from the data file. Similarly, one can extract data of other variables (e.g., longitude) that saved in file.

(3) “GFAS_daily_FRE_data.rar”: There are data files that provide daily FRE at 1° resolution over northeastern Asia during 2012–2019, which is calculated based on FRP density from GFAS (see details in Section 2). Each file is stored in “.bin” format, and a structure with three structure variables are saved in file, namely “FRE”, “lon”, and “lat”. The variable “FRE” is FRE (unit, MJ) in the 1° x 1° grids, “lon” and “lat” are longitude and latitude in decimal degrees of grid center. All structure variables are two-dimensional arrays, and each of them contains 35 (column) x 15 (row) elements. For those grids without fire observation, the missing value of “0.0″ is set for FRE. Likewise, users can easily extract data in data file using methods that they are familiar with (e.g., via IDL commands).

## 2 Experimental Design, Materials and Methods

The top-down NO_x_ fire emission dataset we developed was computed using the combination of NO_x_ EC derived by [1], FRP density from the public dataset GFAS (Global Fire Assimilation System) [2], active fire detections [3] from the sensor VIIRS (Visible Infrared Imaging Radiometer Suite) on Suomi-NPP satellite, and the land cover map [4] from the public platform C3S CDS (Copernicus Climate Change Service, Climate Data Store). For each day during 2012–2019, we calculated for each 1° x 1 ^o^ grid the total NO_x_ fire emission using Equation as follows:

(1) $$NO_{x}=\sum EC_{i}\;*\;\alpha_{i}\;*\;\text{FR}E_{\text{GFAS}}$$

where “*i* = 1, 2, 3″ indicate the three fire categories: low-biomass vegetation (including cropland, grassland, and herbaceous vegetation) fires and forest fires if ≥ 75% of FRP is contributed by that category, or the other vegetation fires. EC values of 1.19 g/MJ for low-biomass vegetation fires, 0.41 g/MJ for forest fires, and 0.46 g/MJ for other vegetation fires [1] were applied in the equation. α is FRP fraction of the *i*th fire category, calculated from Eq. (2), which is used as partition coefficient of FRE_GFAS_ in Eq. (1).

(2) $$\alpha_{i}=\frac{\text{FR}P_{i}}{\sum \text{FR}P_{i}}$$

In Eq. (2), “i” represents the same fire categories used in Eq. (1), FRP (unit, MW) is fire radiative power of active fire detections from the VIIRS 375-m active fire product VNP14IMG [3], which is publicly available from LAADS DAAC (Level-1 and Atmosphere Archive & Distribution System Distributed Active Archive Center, https://ladsweb.modaps.eosdis.nasa.gov/). In the above processing, we used the 300-m annual land cover map at fire year from the public platform C3S CDS (https://cds.climate.copernicus.eu/) to identify the vegetation type of VIIRS fires.

(3) $$FRE_{\text{GFAS}}=\sum_{j=1}^{j=n}\text{FRP}\_\text{densit}y_{j}*A_{j}*\delta t$$

In Eq. (3), FRE_GFAS_ (unit, MJ) is the FRE calculated using daily FRP density (unit, MW/m^2^) at a spatial resolution of 0.1° from the public dataset GFAS (https://apps.ecmwf.int/datasets/data/cams-gfas/) in European centre for Medium-Range Weather Forecasts; “n” is the number of GFAS data point within a given 1° x 1° grid; “j” indicate the *j*th GFAS data point; A (unit, m^2^) is the ground area of the GFAS data point, and δt (unit, s) is day length.

## Ethics Statements

Not applicable.

## CRediT authorship contribution statement

**Yuyun Fu:** Conceptualization, Data curation, Investigation, Methodology, Software, Visualization, Writing – original draft, Writing – review & editing. **Rui Li:** Writing – review & editing, Resources, Funding acquisition. **Jiheng Hu:** Investigation, Writing – review & editing. **Yipu Wang:** Writing – review & editing. **Jiawei Duan:** Writing – review & editing.

## Declaration of Competing Interest

The authors declare that they have no known competing financial interests or personal relationships that could have appeared to influence the work reported in this paper.

## Data Availability

Full Top Down NOx Fire Emission (Original data) (Mendeley Data).

## Appendix Supplementary materials

Image, application 1
